# Comparing Fractionated and Single-Fraction Gamma Knife Radiosurgery for Brain Metastases From Non-Small-Cell Lung Cancer With a Focus on Driver Alterations

**DOI:** 10.7759/cureus.41849

**Published:** 2023-07-13

**Authors:** Mariko Kawashima, Atsuya Akabane, Ryuichi Noda, Masafumi Segawa, Sho Tsunoda, Tomohiro Inoue

**Affiliations:** 1 Gamma Knife Center, NTT Medical Center Tokyo, Tokyo, JPN; 2 Department of Neurosurgery, NTT Medical Center Tokyo, Tokyo, JPN

**Keywords:** non-small-cell lung cancer, gamma knife, fractionated radiosurgery, multifraction, brain metastasis

## Abstract

Background

As the overall survival in non-small-cell lung cancer has increased, safer, long-term treatments for brain metastases are increasingly needed. This study aimed to analyze the outcomes of fractionated and single-fraction gamma knife radiosurgery for brain metastases from non-small-cell lung cancer, focusing on driver alteration status.

Methodology

Patients who underwent gamma knife radiosurgery as their first local treatment for brain metastases from non-small-cell lung cancer between May 2018 and December 2021 at our institution were retrospectively enrolled.

Results

Among the 98 patients (287 lesions), 45 (130 lesions) harbored driver alterations, including epidermal growth factor receptor mutations in 35 patients and anaplastic lymphoma kinase or ROS1 rearrangement in 10 patients. Overall, 64 and 34 patients underwent single-fraction and fractionated radiosurgery (3-15 fractions), respectively. Large tumor size was a risk factor for recurrence, while fractionated radiosurgery (subdistribution hazard ratio (sHR) = 16.47; confidence interval (CI) = 3.58-75.77; p < 0.001) and small tumor size (sHR = 1.15; CI = 1.04-1.28; p = 0.008) independently protected against radiation necrosis. In the case-matched analyses, the cumulative radiation necrosis rates were significantly lower in the fractionated group than in the single-fraction group among all lesions (p = 0.017) and among those with driver alterations (p = 0.046), whereas no significant difference was confirmed among wild-type lesions (p = 0.382).

Conclusions

Fractionated gamma knife radiosurgery may be an alternative therapeutic approach for reducing the risk of radiation necrosis, particularly for patients with driver alterations, even when the tumors are small. Further research is necessary to determine the optimal indications for fractionated gamma knife radiosurgery and fractionation methods.

## Introduction

Brain metastases (BMs) frequently occur in patients with non-small-cell lung cancer (NSCLC), especially in those harboring driver alterations [1]. The most frequent alterations among Asians are epidermal growth factor receptor (EGFR) mutations, which have been detected in up to 60-70% of lung adenocarcinomas in Asian never-smokers, in contrast to 10-15% of Caucasian patients [2]. Targeted therapies, including treatment with EGFR-tyrosine kinase inhibitors (TKIs) and anaplastic lymphoma kinase (ALK)-TKIs, have dramatically improved patient survival over the past decade [3-6]. Most importantly, new-generation TKIs, such as osimertinib and lorlatinib, have shown a high ability to penetrate the blood-brain barrier, allowing a delay in local therapy in some cases [7,8]. Nevertheless, many patients eventually require local therapy for intracranial progression owing to acquired drug resistance during the course of their disease. Stereotactic radiosurgery (SRS) is used in patients, especially in those with up to 10 BMs, to address concerns regarding the possibility of neurocognitive decline caused by whole-brain radiotherapy (WBRT) [9-11]. Thus, SRS plays a pivotal role in the management of BMs from NSCLC.

While the gamma knife has been widely acknowledged as the most precise device for single-fraction radiosurgery and has shown a high level of long-term effectiveness and safety, fractionated gamma knife radiosurgery (fGKRS) is a relatively recent development, with its latest model, the Leksell Gamma Knife Icon (Elekta, Stockholm, Sweden), being introduced in the late 2010s [12]. Although the effectiveness of fractionated radiosurgery for BMs larger than 2-3 cm in diameter has been widely acknowledged, the optimal indications for fGKRS, other than large tumor size, have not yet been fully elucidated. Meanwhile, many researchers have examined the impact of oncogenic driver alterations and the use of TKIs on SRS outcomes [6,13,14], with several studies suggesting that patients with NSCLC-BMs who harbor driver alterations and receive TKI therapy are susceptible to radiation necrosis following SRS [15-18]. Therefore, such patients require cautious care. As many patients with BMs are referred to our tertiary hospital, we started using fGKRS in 2018 and gradually broadened its scope in our search for a safer therapeutic option than the ones being used so far. Therefore, in this study, we aimed to analyze the preliminary outcomes of radiosurgery for NSCLC-BMs from the beginning of the use of fGKRS at our center in the era of central nervous system-penetrant TKIs.

## Materials and methods

Patient selection

This retrospective study was approved by the institutional review board of NTT Medical Center Tokyo (IRB number: 22-93) and was conducted in accordance with the tenets of the Declaration of Helsinki (revised version in 2013). Written informed consent was obtained from all patients.

We enrolled consecutive patients with BMs from NSCLC who underwent gamma knife radiosurgery as their initial intracranial treatment at our institution between May 2018, when we began using the Leksell Gamma Knife Icon, and December 2021. We excluded patients with unknown status of genetic alterations, those with tumors smaller than 5 mm in diameter, those who underwent staged radiosurgery, those who underwent postoperative radiosurgery only for the tumor bed, and patients whose post-treatment data could not be obtained. A total of 287 lesions from 98 patients were included in this study.

Treatment procedures

All procedures were performed using a Leksell Gamma Knife Icon. fGKRS was originally selected for patients with large tumors (≥20 mm in diameter) and tumors in eloquent locations, and in these patients, it was also performed on their other small tumors during the same treatment. fGKRS was generally performed with five fractions, but 10 or more fractions were applied for large tumors, exceeding 10 mL, or in patients with many lesions. In addition, the number of fractions was increased for tumors in the brainstem and near the optic apparatus. The number of fractions was sometimes adjusted because of weekends or national holidays. Standardized planning magnetic resonance imaging (MRI) and computed tomography (CT) were performed within three days before treatment. For patient immobilization, the Leksell G frame or device-specific thermoplastic mask was used in patients receiving single-fraction gamma knife radiosurgery (sGKRS), whereas the mask alone was used in those receiving fGKRS. Stereotactic cone-beam CT was performed with head fixation using the frame or mask. The prescribed dose in a single fraction ranged from 16 to 22 Gy, while the biologically effective doses in fGKRS ranged from 40 to 60 Gy and were determined using the linear-quadratic model with an alpha-beta ratio of 10. For fGKRS, irradiation was performed on consecutive weekdays. Interfractional evaluation was performed when the treatment continued for longer than one week, and re-planning was performed, if necessary, as detailed previously [19]. All treatment plans were meticulously formulated by the same senior physician (AA).

Clinical outcomes

After treatment, the patients were observed every one to three months at the referring hospitals or our institution. The endpoints of this study were overall survival (OS), neurological death, tumor recurrence, radiation necrosis, and radiation-induced adverse events (RAEs). Neurological death was defined as death due to intracranial tumor progression, whereas systemic death was attributed to extracranial disease progression. Local tumor recurrence was defined as ≥20% enlargement in the diameter of the target lesion characterized by contrast enhancement in comparison with the images obtained at the time of treatment. Furthermore, tumor recurrence was distinguished from radiation necrosis according to histopathological assessments or imaging findings such as a T1/T2 mismatch on MRI, arterial spin labeling magnetic resonance perfusion imaging, dynamic-susceptibility contrast-enhanced magnetic resonance perfusion imaging, and 11C-methionine or 18F-fluorodeoxyglucose positron emission tomography. The response to corticosteroids was also utilized for judgment. Imaging results were also correlated with observed clinical deterioration, as defined by the Common Terminology Criteria for Adverse Events (CTCAE) version 5.0, and events with CTCAE grade 2 or worse were judged as RAEs.

Statistical analysis

Patients’ baseline characteristics and treatment parameters were summarized and compared using the Mann-Whitney test for continuous variables and Fisher’s exact test for categorical variables. The OS rates were calculated using the Kaplan-Meier method and compared using the log-rank test. The cumulative neurological death rate was calculated using Gray’s test, where systemic death was regarded as a competing event. The prognostic factors for local tumor recurrence and radiation necrosis were evaluated using a competing risk analysis model, the Fine-Gray proportional hazards model, where subsequent WBRT for intracranial progression and the patient’s death were regarded as competing events for both tumor recurrence and radiation necrosis. To reduce the effects of selection bias and potential confounding factors, we performed propensity score matching. sGKRS and fGKRS groups were matched in a 1:1 ratio with a caliper of 0.2 standard deviations of the logit of the estimated propensity score using logistic regression to control covariates such as age, sex, extracranial disease status, driver alteration status, number of brain metastases, and tumor volume. After matching, the covariates were compared using the Mann-Whitney test for continuous variables and Fisher’s exact test for categorical variables. The balance among the covariates was assessed using standardized differences. A standardized difference of ≤0.1 was considered an indicator of a well-balanced result. After matching, the cumulative rates of tumor recurrence and radiation necrosis were compared for the sGKRS and fGKRS groups using Gray’s test. The time interval analyzed was from the date of radiosurgery until the occurrence of each event. All statistical analyses were performed using SPSS Statistics (version 28.0, IBM Corp., Armonk, NY, USA) and EZR (Saitama Medical Center, Jichii Medical University), which is a graphical user interface for R version 4.1.2 (The R Foundation for Statistical Computing, Vienna, Austria).

## Results

The clinical characteristics of the entire cohort, driver alteration type, and wild type are summarized in Table 1. The cohort included 45 patients with 130 lesions who harbored the following oncogenic alterations: EGFR mutations in 35 patients with 113 lesions, ALK rearrangement in five patients with seven lesions, and ROS1 rearrangement in five patients with 10 lesions. Concurrent TKI administration within the four weeks before and after starting radiosurgery was performed in 34 patients, accounting for 76% of patients with driver alterations. The median volume of the largest tumor in each patient was higher in the driver alteration type than in the wild type (p = 0.002). The median volume of all tumors was higher in the driver alteration type than in the wild type as well (p = 0.010). sGKRS was performed in 64 patients, while fGKRS (median number of fractions = 5; range = 3-15) was performed in 34 patients. The median prescribed dose and maximum dose among the lesions treated using sGKRS were 21 Gy (interquartile range (IQR) = 20-22 Gy) and 31 Gy (IQR = 29-33 Gy), respectively. The most frequently employed fractionation regimen in the fGKRS group was 32.5-35 Gy delivered in five fractions, comprising 56% of patients treated with fGKRS. There were no significant differences between patients who underwent sGKRS and those who underwent fGKRS in terms of age, sex, driver alteration type, Karnofsky performance status, uncontrolled extracranial lesion status, and number of BMs. However, the median volume of the largest tumor in each patient was higher in the fGKRS group than in the sGKRS group. The median volume of all tumors was also higher in the fGKRS than in the sGKRS group.

**Table 1:** Baseline characteristics of the included patients and tumors. *: Statistically significant (p < 0.05). IQR = interquartile range; KPS = Karnofsky performance status; n = number of patients; N = number of brain metastases

Variables	Entire cohort	Driver alteration type	Wild type	P-value
	(n = 98; N = 287)	(n = 45; N = 130)	(n = 53; N = 157)	
Median age (years)	68 (IQR 60–75)	68 (IQR 58–76)	69 (IQR 64–75)	0.523
Male sex, n (%)	58 (59)	17 (37.8)	41 (77.4)	<0.001
KPS, n (%)				0.193
≤ 70	16 (17)	6 (13)	11 (21)	
80	21 (22)	7 (16)	14 (26)	
90–100	60 (61)	32 (71)	28 (53)	
Median number of brain metastases	3 (IQR 1–7)	3 (IQR 1–7)	2 (IQR 1–7)	0.636
Uncontrolled extracranial cancer, n (%)	45 (46)	17 (38)	28 (53)	0.158
Number of fractions, n (%)				0.486
1	64 (65)	32 (71)	32 (60)	
3–5	20 (20)	7 (16)	13 (25)	
6–15	14 (14)	6 (13)	8 (15)	
Median volume of the maximum tumor per patient (mL)	0.8 (IQR 0.39–3.6)	0.5 (IQR 0.3–2.0)	1.5 (IQR 0.6–5.1)	0.002
Median tumor volume (mL)	0.2 (IQR 0.09–0.7)	0.2 (IQR 0.1–0.6)	0.3 (IQR 0.1–1.0)	0.010

Overall survival and neurological death

The median observation period after treatment was 15.5 months (IQR = 7.9-29.0 months). The OS in patients with driver alterations was 97% at one year and 88% at two years, which was significantly higher than that in the wild type (78% at one year and 70% at two years; p = 0.007). In total, 19 patients died during the follow-up period (median follow-up duration = 10 months). Among them, 15 patients died of systemic disease, while the remaining four deaths were categorized as neurological deaths, which included tumor recurrence of the irradiated lesion in two patients and leptomeningeal disease in two patients. The cumulative neurological death rate in the entire cohort was 0.9% at one year and 4.1% at two years, which was not statistically different between the patients treated with sGKRS and fGKRS (p = 0.847). Additionally, the cumulative neurological death rate in driver alteration type was not different between the sGKRS and fGKRS groups (p = 0.236).

Local tumor recurrence and radiation necrosis

After treatment, 25 lesions (14 in the sGKRS and 11 in the fGKRS group) were regarded as tumor recurrence cases. Recurrence was managed with resection for eight lesions and repeat fGKRS for 13 lesions. In addition, TKI therapy was introduced in two recurrent lesions, while no treatment was implemented for two other lesions due to systemic deterioration. Large tumor size was a risk factor for tumor recurrence in all lesions (subdistribution hazard ratio (sHR) = 1.13; 95% confidence interval (CI) = 1.05-1.20; p < 0001) and in lesions with driver alterations (sHR = 5.01; 95% CI = 1.77-14.20; p = 0.002) in the multivariate analysis (Table 2). Radiation necrosis was observed in 14 tumors (13 treated with sGKRS and in one treated with fGKRS). Corticosteroids and/or vitamin E agents were administered for 11 lesions and bevacizumab was administered for two lesions, while one lesion treated with sGKRS was resected. As a result, RAEs were confirmed in 12 patients; among them, one patient developed intratumoral hemorrhage five months after radiosurgery. No patients experienced adverse events with CTCAE grade >2 except one patient with an adverse event of grade 3. Multivariate analysis demonstrated that advanced age (sHR = 1.06; 95% CI = 1.01-1.12; p = 0.034), a large tumor size (sHR = 1.15; 95% CI = 1.04-1.28; p = 0.008), and sGKRS (sHR = 16.47; 95% CI = 3.58-75.77; p < 0.001) were significant risk factors for radiation necrosis in the entire cohort, whereas a large tumor size (sHR = 1.49; 95% CI = 1.12-1.97; p = 0.006) and sGKRS (sHR = 12.03; 95% CI = 3.73-38.83; p < 0.001) were risk factors in lesions with driver alterations (Table 2).

**Table 2:** Multivariate analyses of factors associated with local tumor recurrence and radiation necrosis *: Statistically significant (p < 0.05). CI = confidence interval; fGKRS = fractionated gamma knife radiosurgery; HR = hazard ratio; N = number of brain metastases; sGKRS = single-fraction gamma knife radiosurgery; sHR = subdistribution hazard ratio

	Tumor recurrence		Radiation necrosis	
	P-value	sHR (95% CI)	P-value	sHR (95% CI)
Overall (N = 287)				
Age (years, continuous)	0.880	1.00 (0.96–1.05)	0.034*	1.06 (1.01–1.12)
Male (vs. female)	0.340	1.58 (0.62–4.00)	0.290	0.57 (0.20–1.60)
Tumor volume (mL, continuous)	<0.001*	1.13 (1.05–1.20)	0.008*	1.15 (1.04–1.28)
sGKRS (vs. fGKRS)	0.790	0.89 (0.39–2.05)	<0.001*	16.47 (3.58–75.77)
Driver alteration type (N = 130)				
Age (years, continuous)	0.610	0.98 (0.92–1.05)	0.081	1.08 (0.99–1.18)
Male (vs. female)	0.290	2.08 (0.54–8.00)	0.760	1.26 (0.30–5.29)
Tumor volume (mL, continuous)	0.002*	5.01 (1.77–14.20)	0.006*	1.49 (1.12–1.97)
sGKRS (vs. fGKRS)	0.240	2.16 (0.60–7.77)	<0.001*	12.03 (3.73–38.83)

Outcomes of the matched cohorts

**Figure 1:**
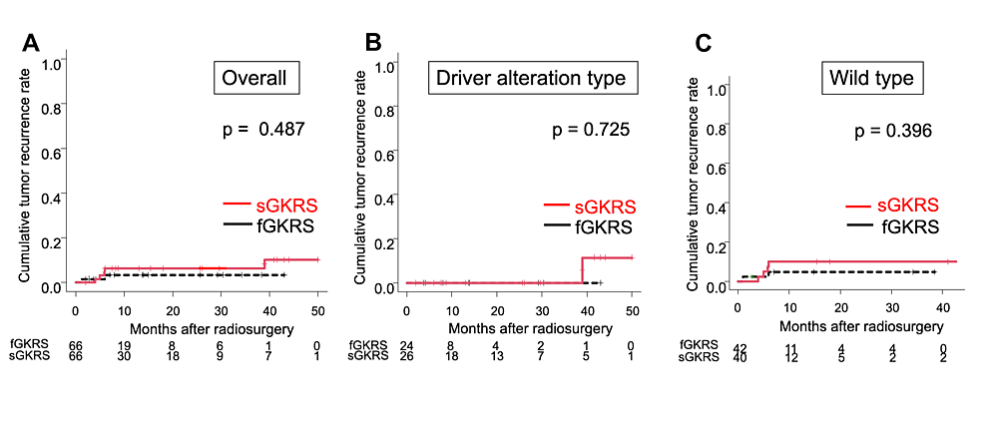
Cumulative tumor recurrence rates of sGKRS and fGKRS in the case-matched lesions. A: All lesions. B: Lesions with driver alterations. C: Wild-type lesions. sGKRS = single-fraction gamma knife radiosurgery; fGKRS = fractionated gamma knife radiosurgery

**Figure 2:**
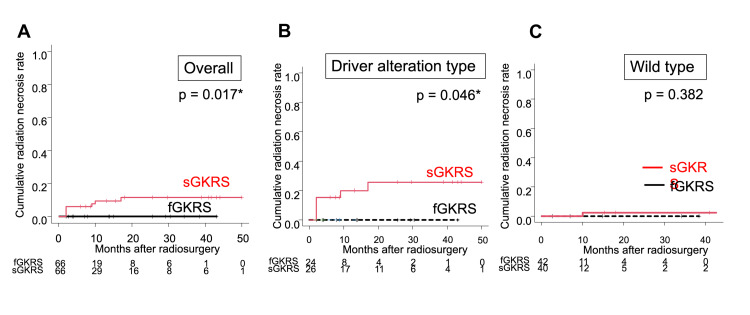
Cumulative radiation necrosis rate of sGKRS and fGKRS in the case-matched lesions. A: All lesions. B: Lesions with driver alterations. C: Wild-type lesions. *: Statistically significant (p < 0.05). sGKRS = single-fraction gamma knife radiosurgery; fGKRS = fractionated gamma knife radiosurgery

**Figure 3:**
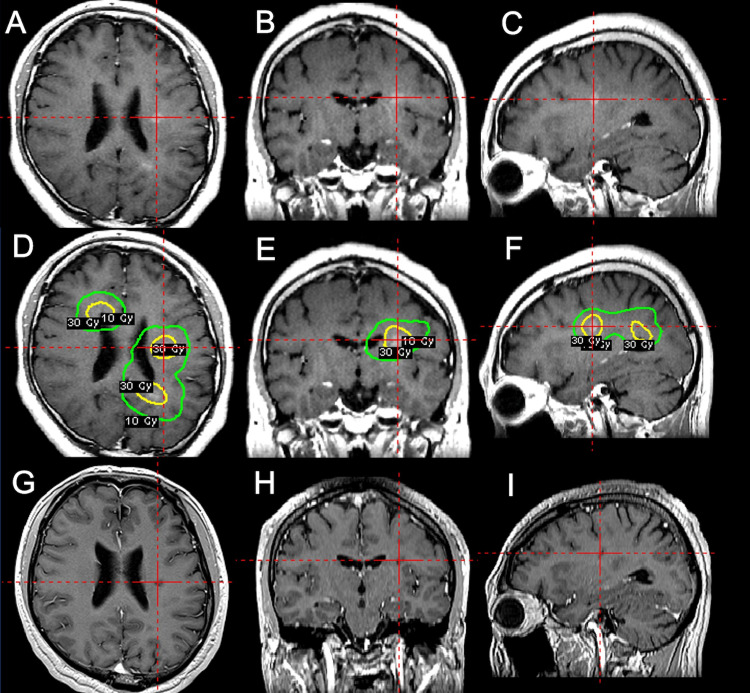
An illustrative case. A 35-year-old man with ALK-positive lung adenocarcinoma with a 10-year history of ALK-TKI administration developed intracranial contrast-enhanced lesions 16 months before fGKRS that gradually enlarged. The radiographical findings including 11C-methionine-positron emission tomography were consistent with brain metastases. A, B, C: Gadolinium-enhanced T1-weighted MRI before fGKRS. D, E, F: The equivalent images in the planning of fGKRS. The tumors were treated with a prescription dose of 30 Gy (maximum 40 Gy) in 10 fractions. Because the tumors were poorly circumscribed with contrast enhancement, we set enough margin for the enhanced lesion. The yellow line indicates the prescription dose 30 Gy-line for the tumors, and the green line indicates the 10 Gy-line. G, H, I: Images obtained at 10 months after fGKRS. The enhanced lesions completely disappeared and no brain edema around them was confirmed without neurological symptoms. A, D, G: axial images, B, E, H: coronal images, C, F, I: sagittal images. ALK = anaplastic lymphoma kinase; TKI = tyrosine kinase inhibitor; fGKRS = fractionated gamma knife radiosurgery

We created matched cohorts of 66 lesions each for sGKRS and fGKRS and compared their characteristics (Table 3). After matching, the standardized differences of all the covariates were ≤0.1, indicating that there was lower bias compared to the unmatched cohorts. No significant difference was observed in the cumulative tumor recurrence rates between the sGKRS and fGKRS groups for all lesions (p = 0.487), lesions with driver alterations (p = 0.725), and wild-type lesions (p = 0.396) (Figure 1). In contrast, the cumulative radiation necrosis rates were significantly higher in the sGKRS group than in the fGKRS group for all lesions (p = 0.017) and those with driver alterations (p = 0.046), but no significant difference was confirmed for wild-type lesions (p = 0.382) (Figure 2). An illustrative case harboring a driver alteration is shown in Figure 3.

**Table 3:** Balance of covariates between the sGKRS and fGKRS groups before and after matching. *: Statistically significant (p < 0.05). BMs = brain metastases; fGKRS = fractionated gamma knife radiosurgery; KPS = Karnofsky performance status; N = number of brain metastases; SD = standard deviation; sGKRS = single-fraction gamma knife radiosurgery

Covariate	Before matching (N = 287)				After matching (N = 132)			
	sGKRS (N = 159)	fGKRS (N = 128)	P-value	Standardized difference	sGKRS (N = 66)	fGKRS (N = 66)	P-value	Standardized difference
Age, mean ± SD (years)	64.41 ± 11.5	68.86 ± 10.99	0.001*	0.396	67.1 ± 12.14	66.6 ± 11.84	0.712	0.038
Male, N (%)	112 (71)	52 (41)	<0.001*	0.604	38 (58)	41 (60)	0.723	0.093
Number of BMs ≥5, N (%)	98 (62)	94 (73)	0.043*	0.292	45 (68)	47 (71)	0.850	0.066
Controlled extracranial disease, N (%)	71 (45)	61 (48)	0.635	0.060	21 (36)	24 (36)	0.714	0.096
Driver alteration type, N (%)	69 (43)	62 (48)	0.477	0.086	26 (39)	24 (36)	0.858	0.062
Tumor volume, mean ± SD (mL)	0.50 ± 0.77	1.91 ± 3.70	0.018*	0.528	0.68 ± 1.00	0.68 ± 1.24	0.736	0.001

## Discussion

Our findings suggest that fGKRS is as effective as sGKRS in controlling NSCLC-BMs (Table 2, Figure 1) and that the rates of radiation necrosis were significantly lower in the lesions treated with fGKRS than in those treated with sGKRS (Table 2, Figures 2A, 2B). These results were demonstrated by both multivariate and case-matched analyses and have not been previously discussed in the literature.

Historically, staged radiosurgery or linear accelerator-based fractionated radiotherapy was introduced as a mitigation strategy for large BMs [20-24]. Fractionated radiosurgery or radiotherapy has been reported to reduce the risk of radiation necrosis without worsening local control for large tumors in comparison with single-fraction radiosurgery using a compromised dose. Currently, the most prevalent fractionation method is 27 Gy in three fractions and 30-35 Gy in five fractions for tumors >20 mm in diameter [20,21]. However, evidence of an optimal fractionation scheme is still lacking in comparison with sGKRS. In addition, other indications for fractionated radiosurgery, aside from large tumors, have scarcely been investigated, while it has been applied to lesions close to eloquent locations and previously irradiated lesions as a pragmatic approach [25].

Previous studies have suggested that cancer cells with driver alterations can be susceptible to radiation and that TKIs can act as radiosensitizers [26-28]. Additionally, the incidence of radiation necrosis in patients with driver alterations and TKIs was reported to be higher than that in those without such alterations and agents [15-18]. Our study also demonstrated that fGKRS was a protective factor against radiation necrosis, independent of the tumor size. In clinical practice, we sometimes encounter NSCLC-BMs that are poorly circumscribed with contrast enhancement, particularly among patients who had previously received TKIs, leading us to speculate that TKIs may obscure the boundaries of BMs to some extent, as illustrated in Figure 3. In addition, the use of the gamma knife enables a steep dose gradient inside and outside the target, which may result in excessive doses being administered to normal brain tissue in patients having tumors with ambiguous boundaries, which can be a disadvantage of single-fraction irradiation. With targeted therapies, especially third-generation TKIs, patients can now live longer than they could previously [3]. On the other hand, they are at an increased risk of developing radiation necrosis in the long run. Therefore, we believe that the threshold of applying fGKRS in terms of tumor size should be lowered for safer and better intracranial outcomes.

Indeed, radiation necrosis seldom requires urgent treatment in its early stages, and with regular patient surveillance, clinicians can gain time to devise and modify treatment strategies. Nonetheless, the risk of radiation necrosis should not be underestimated. Treatment often involves medical interventions, such as corticosteroids, which can cause various adverse effects with long-term use and even narrow the window of opportunity for systemic therapies. Other management strategies for radiation necrosis include the use of anti-vascular endothelial growth factor (VEGF) agents such as bevacizumab, which have been shown to be effective [29]. However, anti-VEGF agents may have to be occasionally discontinued due to their adverse effects. Alternatively, MRI-guided laser interstitial thermal therapy has been reported to be effective in controlling radiation necrosis, particularly in the United States [30]; however, it is currently unavailable in Japan. Considering these aspects, if the burden on the patient is acceptable, GKRS might be safer than sGKRS, even for small tumors.

This study has several limitations as it represents preliminary results from a single institution. First, the retrospective nature of the study and the relatively small sample size may have introduced selection biases, despite efforts to alleviate them through propensity score matching. Second, not all patients with driver alterations received TKIs concurrently with radiosurgery. Nonetheless, TKIs must have been administered or will eventually be administered for patients with driver alterations at some point in the clinical course of the disease, contributing to their extended survival. Third, among patients receiving fGKRS, the fractionation scheme was not homogenous and data on the optimal fraction and dose for each case are still lacking. Dose escalation in fGKRS can be acceptable considering the low rate of radiation necrosis in this study, particularly for patients without driver alterations. Fourth, in recent years, immune checkpoint inhibitors have been used in many cases without driver gene alterations, which may have affected the risk of radiation necrosis. Fifth, in some cases where replanning was performed during the irradiation period, there was some dissociation between the recorded tumor volume and the actual irradiated tumor volume, which could be considered a bias. Despite these limitations, and in the absence of other studies with a similar perspective, we suggest that fGKRS might be preferable to sGKRS in terms of local control and radiation necrosis, particularly for NSCLC-BM patients with driver alterations, producing an impetus for further clinical research.

## Conclusions

In this retrospective study of NSCLC-BMs, including relatively small tumors, fGKRS resulted in good local control and was more protective against radiation necrosis than sGKRS, especially among patients with driver alterations. Given their high OS, the potential benefit of fGKRS might be significant in patients with driver alterations. Further studies are needed to explore the optimal indications for fGKRS and the fractionation schemes for NSCLC-BMs.
